# Antiplasmodial Activity of Probiotic *Limosilactobacillus fermentum* YZ01 in *Plasmodium berghei* ANKA Infected BALB/c Mice

**DOI:** 10.1155/jotm/6697859

**Published:** 2024-12-12

**Authors:** Timothy Bamgbose, Afshana Quadri, Isa O. Abdullahi, Helen I. Inabo, Mohammed Bello, Lokesh D. Kori, Anupkumar R. Anvikar, José de la Fuente, Elianne Piloto-Sardiñas, Alejandro Cabezas-Cruz

**Affiliations:** ^1^ICMR-National Institute of Malaria Research, Sector 8, Dwarka, New Delhi, India; ^2^Department of Microbiology, Ahmadu Bello University, Samaru Zaria, Kaduna, Nigeria; ^3^Department of Veterinary Public Health and Preventive Medicine, Ahmadu Bello University, Samaru Zaria, Kaduna, Nigeria; ^4^SaBio Instituto de Investigación en Recursos Cinegéticos IREC-CSIC-UCLM-JCCM, Ronda de Toledo s/n, Ciudad Real 13005, Spain; ^5^Department of Veterinary Pathobiology, Center for Veterinary Health Sciences, Oklahoma State University, Stillwater 74078, Oklahoma, USA; ^6^Direction of Animal Health, National Center for Animal and Plant Health, Carretera de Tapaste y Autopista Nacional, Apartado Postal 10, San José de las Lajas 32700, Mayabeque, Cuba; ^7^ANSES, INRAE, Ecole Nationale Vétérinaire d'Alfort, UMR BIPAR, Laboratoire de Santé Animale, Maisons-Alfort F-94700, France

**Keywords:** cytokines, gut microbiome, immunoglobulin, *Lactobacillus*, malaria, parasitaemia, *Plasmodium*, probiotics

## Abstract

Malaria remains a significant global health challenge, with the deadliest infections caused by *Plasmodium falciparum*. In light of the escalating drug resistance and the limited effectiveness of available vaccines, innovative treatment approaches are urgently needed. This study explores the potential of the probiotic *Limosilactobacillus fermentum* YZ01, isolated from traditionally fermented kindirmo milk, to modify host responses to *Plasmodium berghei* ANKA infection. Twenty-five male BALB/c mice were grouped and administered various treatments, including probiotic-enriched yogurt alone or in combination with antibiotics. Parameters assessed included gut lactic acid bacteria (LAB) composition, parasitaemia progression, survival rates, and immune response dynamics over a 21-day postinfection period. The probiotic treatment significantly altered gut microbiota, evidenced by increased LAB counts and modulated immune responses, notably enhancing IgM and IL-4 production while reducing IFN-*γ* levels. Mice receiving prolonged probiotic treatment exhibited delayed parasitaemia onset, reduced mortality rates, and a more robust immune response compared to control groups. These outcomes suggest that probiotic intervention not only tempers the pathological effects of malaria but also enhances host resilience against infection. This study underscores the role of gut microbiota in infectious disease pathogenesis and supports probiotics as a promising adjunct therapy for malaria management.

## 1. Introduction

*Plasmodium*, a protozoan parasite transmitted by the female *Anopheles* mosquito, is the causative agent of malaria, one of the deadliest infectious diseases globally. In humans, *Plasmodium falciparum* is responsible for the most lethal forms of the disease. According to the World Malaria Report 2023 [1], there were approximately 249 million cases of malaria and 608,000 deaths worldwide, underscoring the significant health burden posed by this parasite. Particularly vulnerable are children under 5 years of age, who are at greater risk due to underdeveloped immune system and the waning of maternal immunity [2, 3], whereas adults may develop partial immunity after repeated exposure, mitigating the severity of the disease [4–6].

Current strategies to combat this health menace include several antimalarial drugs, with the Artemisinin-based Combination Therapy (ACT) being the primary treatment [7]. However, the emergence of drug-resistant strains of the parasite presents a formidable challenge, jeopardizing effective control and treatment efforts [8, 9]. Vector control remains the best preventive approach [7, 10], while vaccines in different developmental stages give hope in containing *Plasmodium* transmission [11, 12]. The role of the immune system in the pathophysiology of malaria is critical, as it is influenced by bioactive parasite products that stimulate both proinflammatory and counter-regulatory cytokine responses by innate and adaptive immune cells [13–15]. This complexity opens up the potential for novel approaches such as the use of commensal microorganisms to modulate the immune response.

In this context, probiotics—typically Gram-positive bacteria derived from the gut microflora—have emerged as a promising alternative [16–20]. Probiotics are not only known for their ability to stimulate the immune system but also for their potential to modulate the gut microbiome with beneficial effects [21]. Interactions between probiotics and intestinal immune cells can enhance immune homeostasis and increase antibody production [21, 22]. Research has shown that the composition of the gut microbiota may influence the severity of malaria infection in animal models [23–25] and clinical settings [26–28], with certain probiotic strains potentially reducing disease severity through effects on gut barrier integrity, cytokine production, and overall immune modulation. Notably, alterations in the gut microbiota composition have been linked to various diseases, including malaria [29]. Moreover, the broader implications of probiotics on health extend to influencing the gut-brain axis, which could impact the neurological symptoms often associated with severe cases of malaria [20, 29, 30].

Among the various probiotic strains, *Limosilactobacillus fermentum* has emerged as a candidate of interest due to its notable immunomodulatory properties and ability to influence gut microbiota composition positively [31–33]. Specifically, *L. fermentum* YZ01, isolated from traditionally fermented kindirmo milk [34], has demonstrated potential in modulating host immune responses [35]. Previous studies have indicated that strains of *L. fermentum* can stimulate both innate and adaptive immunity, leading to increased production of antibodies and regulation of cytokine profiles [36, 37]. Given these attributes, we hypothesize that oral administration of *L. fermentum* YZ01 could modulate the immune system and gut microbiota in a way that reduces the severity of malaria infection. The objective of this study is to investigate the antiplasmodial effects of *L. fermentum* YZ01 in a murine model by assessing its impact on parasitaemia progression, survival rates, and immune response in mice infected with *Plasmodium berghei* ANKA. This research aims to provide insights into how probiotics might contribute to malaria management, potentially offering a novel adjunctive strategy in the fight against this devastating disease.

## 2. Materials and Methods

### 2.1. Formulation of Probiotic Yoghurt

Probiotic yogurt was formulated in the laboratory using the lactic acid bacteria (LAB) strain *Limosilactobacillus fermentum* YZ01 (Accession Number MT464039) [35]. Briefly, 100 mL of fresh cow milk were heated at 63°C for 30 min and cooled to 40°C. The LAB strain, which had been washed three times with PBS and adjusted to a concentration of 10^9^ Colony Forming Units (CFU)/mL, was then inoculated into the milk and incubated at 37°C for 8 h before cooling in a refrigerator at 4°C overnight for complete coagulation. The experimental mice were administered 200 μL of the formulated yoghurt, containing between 10^8^ and 10^10^ CFU/mL, throughout the feeding period.

### 2.2. Experimental Animal and Housing

Male BALB/c mice, aged 6–8 weeks and weighing an average of 20 g, were bred at the in-house animal facility of the National Institute of Biologicals (NIB, NOIDA 824/GO/RBiBt/S/04/CPCSEA). These mice were procured and subsequently transported to the animal house facility at the Indian Council of Medical Research-National Institute of Malaria Research (ICMR-NIMR, No.33/GO/ReBi/S/99/CPCSEA), where they were quarantined for 1 week to acclimatize to the new environment. Animal care and handling were conducted in compliance with the guidelines of the Committee for the Purpose of Control and Supervision of Experiments on Animals (CPCSEA), Government of India. The experimental protocol was approved by the Institutional Animal Ethics Committee (IAEC/NIMR/2020-1/03) of ICMR-NIMR.

Each group of mice was housed in conventional wire grid-topped cages, maintained at stable room temperature of 25°C ± 2°C, and subjected to a 12:12 h light/dark cycle with a relative humidity of 70%–80%. The mice were fed UV-radiated diet pellets specifically formulated for laboratory mice, and had access to sterilized water, which was replaced daily.

### 2.3. Grouping and Feeding of Animals

Twenty-five male BALB/c mice were randomly assigned into five groups, with five mice per group (Table 1). Group I (Healthy Group) consisted of uninfected and untreated mice. Group II (control group) comprised mice that were infected but remained untreated. Group III (Group A) included mice fed with 200 μL of the formulated yoghurt containing 10^9^ CFU/mL of *L. fermentum* YZ01, administered daily for 3 weeks. Group IV (Group B) mice were administered 200 μL of the same concentration of formulated yogurt daily for 1 week. Group V (Group C) mice received an initial treatment with the antibiotic Metronidazole (100 μL of 10 mg/kg) for 1 week, followed by daily administration of 200 μL of the formulated yogurt for 2 weeks. The feeding was carried out using oral gavage for adequate delivery of the food formulation.

### 2.4. Experimental Infection of Mice With *Plasmodium berghei* ANKA

The *Plasmodium berghei* strain ANKA, used for the experimental infections, was sourced from ICMR-NIMR Institute's parasite bank, where it is stored cryogenically in liquid nitrogen within infected red blood cells (iRBC). To prepare for infection, the vial containing the iRBC was thawed in a water bath at 37°C to resuscitate the parasites. These parasites were then propagated in donor mice by injecting 100 μL of iRBC, ensuring a continuous passage for use in experiments. The experimental mice were injected intraperitoneally with 1 × 10^5^ (100 μL) of *P. berghei* ANKA iRBC to establish the infection.

### 2.5. Serum Preparation

Blood samples were collected from mice in each group on days 10, 15 and 20 postinfection using the retro-orbital method. For each collection, 100 μL of blood was drawn into a 1.5 mL tube. The collected blood was allowed to rest undisturbed at room temperature for 15 min to facilitate clotting. Subsequently, the tubes were centrifuged at 4000 rpm for 15 min. After centrifugation, the supernatant serum was carefully pipetted into clean 1.5 mL tubes and immediately stored at −20°C for further analysis.

### 2.6. Impact of *L. fermentum* on Infected Experimental BALB/c Mice

#### 2.6.1. Quantification of *Lactobacillus* From Mice Gut

Faecal samples were collected from mice prior to the administration of the formulated yoghurt and then again on the fourth- and seventh-days postfeeding. The samples were homogenized in PBS (1 g in 10 mL), serially diluted tenfold, and subsequently cultured on Man–Rogosa–Sharpe (MRS) agar. Plates were incubated anaerobically at 37°C for 48 h. After incubation, bacterial colonies were counted and the results expressed as logarithmic CFU/mL (log CFU/mL), allowing for the quantitative analysis of *Lactobacillus* levels in the faeces, following protocols established by Luang-In et al. [38].

#### 2.6.2. Determination of Parasitaemia and Survival of Experimental Mice

Blood samples were collected daily from the tail of each mouse from day 2 to 21 postinfection for parasitaemia evaluation. To prepare for analysis, the blood was used to make thin smears on glass slides, which were then fixed and stained with Giemsa stain. The parasitaemia was assessed through microscopic examination, inspecting at least 10 high-power (× 100 magnification) fields per slide. Within these fields, both total red blood cells (tRBCs) and parasitized RBCs (pRBCs) were counted. The percentage of parasitaemia was calculated using the formula:

(1) $$\begin{array}{c} \text{Parasitaemia }\left(\%\right)=\left[\frac{\text{pRBCs}}{\text{tRBCs}}\right]\times 100. \end{array}$$

Additionally, the mortality rate of each experimental group was monitored daily and recorded. Survival data were plotted as survival curves to visually represent the longevity and health outcomes of the mice over the course of the infection.

#### 2.6.3. Evaluation of Immune Response

Serum samples obtained from mice were analysed for immunoglobulins IgM and IgG3, as well as the cytokines interleukin-4 (IL-4) and interferon-gamma (IFN-*γ*), using a precoated FineTest ELISA 96T kit (Wuhan, China, Batch No: MO119F083) following the manufacturer's instructions. Briefly, standards were prepared from stock solutions and serially diluted to a 10^−6^ concentration. Similarly, the serum samples were diluted using a sample solution buffer, with the buffer alone serving as the control (blank wells). Each well of the ELISA plate received 100 μL of the diluted serum, after which the plate was sealed with a foil and incubated at 37°C for 90 min. After incubation, the content was discarded and the plate was washed twice with wash buffer without allowing the wells to dry up completely. In each well, 100 μL of Biotin-labelled antibody that served as the working solution was added to the bottom of the well, covered and incubated at 37°C for 60 min. Afterwards, the plate was washed three times with wash buffer and was allowed to stay in the well for 2 min each time before washing. This was followed by the addition of 100 μL HRP-Streptavidin Conjugate (SABC) into each well and incubated at 37°C for 30 min. The plate was then washed with wash buffer five times with the wash buffer being allowed to stay for 2 min before washing. Subsequently, 90 μL TMB substrate was added into each well and incubated at 37°C in the dark for 10–20 min until an apparent gradient appeared in the standard wells. Finally, 50 μL of 0.1M sulphuric acid stop solution was added into each well and optical density was measured at 450 nm.

### 2.7. Statistical Analysis

Statistical analyses were conducted using GraphPad Prism (version 5.0) and SPSS (version 20). The differences between group means were assessed using one-way Analysis of Variance (ANOVA). A *p* value of less than 0.05 was considered statistically significant.

## 3. Results

### 3.1. Probiotic Yogurt Supplementation Increases LAB in Faecal Samples of BALB/c Mice

**Figure 1:**
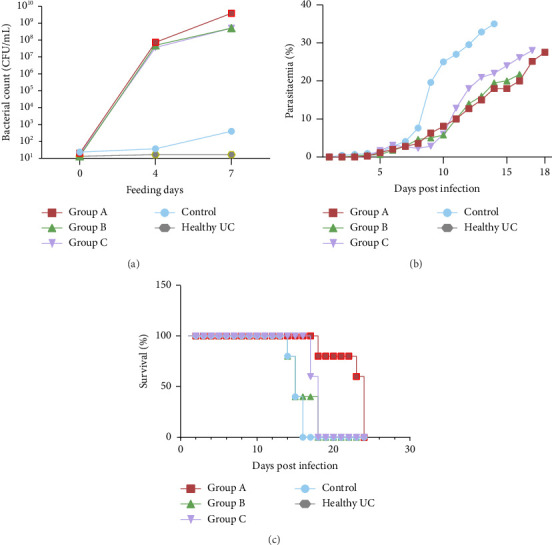
Impact of *Limosilactobacillus fermentum* on infected experimental mice. (a) Lactic acid bacteria count (CFU/mL) in faeces before and after feeding with formulated yoghurt. (b) Percentage of parasitaemia following *P. berghei* infection: Numbers of infected red blood cells (iRBC) were estimated from random 10 fields of microscopic view at 100x oil immersion of thin blood smear. (c) Survival of mice following *Plasmodium* infection: The result is represented as a percentage of survival following mortality in each mouse group. Data are the cumulative result of mouse groups (*n* = 5 in each group). Control-mice infected with *P. berghei* ANKA and untreated, Group A-mice fed with formulated yoghurt for 3 weeks prior to infection with *P. berghei*, Group B-mice fed with formulated yoghurt for 1 week prior to infection with *P. berghei*, Group C-mice were treated with metronidazole for 1 week and fed with formulated yoghurt for 2 weeks prior to infection with *P. berghei* and healthy UC-mice that were uninfected and untreated with formulated yoghurt. Analysis was conducted within 1 week of feeding with formulated yoghurt.

The LAB count in mice faecal samples were analysed prior to feeding and at 4- and 7-day postadministration of the formulated yoghurt. The healthy, unchallenged group maintained a nearly constant LAB population of 1 × 10^1^ CFU/mL. In contrast, the control infected group exhibited a slight increase in LAB counts, which may be attributed to dysbiosis by the infection. Groups A, B and C, which were fed the formulated yoghurt, demonstrated significantly elevated LAB counts ranging from 7 × 10^8^ to 10^9^ CFU/mL after feeding (Figure 1(a)).

### 3.2. Probiotic Intervention Modulates Parasitaemia Dynamics in *Plasmodium*-Infected BALB/c Mice

The parasitaemia was assessed by examining different fields at 1000x magnification under oil immersion from day 2 to day 21 postinfection. The progression of parasitaemia varied over time among the different groups, with the control group having the highest peak; however, there was no parasite clearance observed in this group (Figure 1(b)). By day 10 postinfection, the control group showed an average parasitaemia of 25.0%, while Groups A and B had average levels of 18.0% and 5.7%, respectively. Group C showed a delayed onset of parasitaemia, averaging 6.0% on the same day. By day 15 postinfection, the control group had an average parasitaemia of 36.1%, whereas Group A exhibited 18.1%, Group B 19.5% and Group C 22.9%. All test groups surpassed 20% parasitaemia before mortality occurred; however, the rate of progression varied among them. Group A, which showed the longest survival, allowed for a more extended evaluation of parasitaemia (Figure 1(c)). These findings indicate differential impacts of the treatments on parasitaemia progression and survival rates among the groups.

### 3.3. Prolonged Probiotic Consumption Enhances Survival Rates in *Plasmodium*-Infected BALB/c Mice

Mortality rates postinfection were evaluated and presented as survival curves (Figure 1(c)). The highest mortality was observed in the control infected group, while Group A, which received probiotics for 3 weeks prior to infection, demonstrated the lowest mortality. Groups B and C exhibited similar mortality rates. Specifically, in Group A, 100% of the mice survived until day 18 postinfection, and 60% remained alive at the termination of the experiment. In contrast, by day 18 postinfection, 60% of Group C mice and 40% of both Group B and the control group mice had survived; however, none of the mice these groups survived until the end of the experiment. These findings indicate that prolonged consumption of probiotics positively influences the survival rate in BALB/c mice infected with *P. berghei* ANKA, as evidenced by the extended survival observed in Group A mice administered formulated yogurt 3 weeks before infection.

### 3.4. *Limosilactobacillus fermentum* Modulates Host Immune Response

#### 3.4.1. IgM

**Figure 2:**
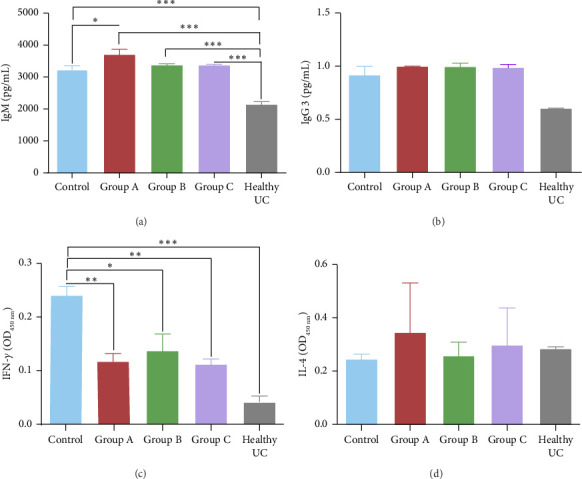
Immune responses in different mouse groups. On day 10, when all infected groups exhibited parasitaemia levels exceeding 5%, immune responses were assessed and compared to a healthy uninfected control (UC) group that received no formulated yoghurt or infection. Measurements included (a) IgM concentration (pg/mL), (b) IgG3 concentration (pg/mL), (c) IFN-*γ* absorbance (OD450), and (d) IL-4 absorbance (OD450). The control group consisted of mice infected with *Plasmodium berghei* ANKA and left untreated. Group A mice were fed formulated yoghurt for 3 weeks prior to infection, Group B for 1 week prior to infection, and Group C for 2 weeks prior to infection, followed by metronidazole treatment for 1 week. The healthy uninfected control group served as a baseline reference. Statistical significance between groups is denoted as follows: ^∗^*p* < 0.05, ^∗∗^*p* < 0.01, ^∗∗∗^*p* < 0.001, while the absence of an asterisk indicates no significant difference.

**Figure 3:**
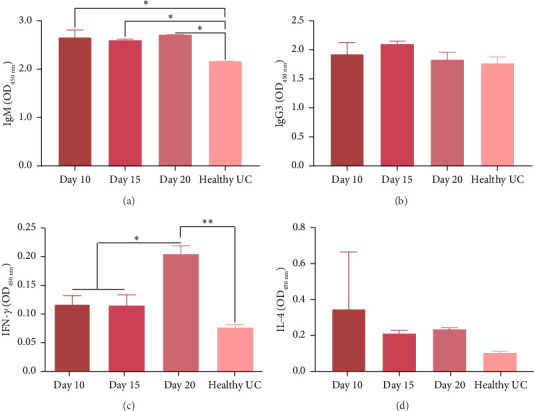
Levels of immune factors in Group A mice. Levels of the immune factors (a) IgM, (b) IgG3, (c) IFN-*γ*, and (d) IL-4 were measured in Group A mice, which had the highest survival rate among the infected groups. The measurements were taken on samples collected on days 10, 15, and 20 postinfection and compared against those in the healthy uninfected control group (healthy UC). All measurements were based on absorbance at OD450. Data were analyzed using one-way ANOVA; bars represent mean ± SD. Statistical significance is indicated as ^∗^*p* < 0.05 and ^∗∗^*p* < 0.01, while the absence of an asterisk indicates no statistical significance.

The levels of IgM were compared among all test groups and the healthy unchallenged group (Figure 2(a)). The control group exhibited elevated IgM levels, likely reflecting the natural immune response of mice to *P. berghei* ANKA infection compared to the healthy group. This suggests that *P. berghei* ANKA infection induces an increase in IgM levels in infected mice. Notably, the probiotic-treated groups demonstrated even higher IgM titres, indicating that probiotics may modulate and enhanced the immune response (Figure 2(a)). In Group A, which showed the highest survival rate, IgM levels were assessed at various days postinfection. As shown in Figure 3(a), there were no significant fluctuations in IgM levels across different days postinfection in Group A, although IgM levels remained consistently higher than those in the healthy unchallenged test group. This sustained elevation of IgM in probiotic-treated mice underscores the potential role of *L. fermentum* in bolstering the host's humoral immune response during *P. berghei* ANKA infection.

#### 3.4.2. IgG3

The levels of IgG3 were compared among all test groups and the healthy unchallenged group. As illustrated in Figure 2(b), the control group exhibited a reduction in IgG3 titre values, whereas Groups A, B and C demonstrated slightly elevated levels compared to the healthy unchallenged group. However, there was no significant difference between the IgG3 titres of the control and the healthy groups, indicating that *P. berghei* infection did not elicit a significant IgG3 immunoglobulin response in mice. Both the control and probiotic-treated groups showed an increase in IgG3 titres, which could be an indication of the natural immune response; however, these changes were not statistically significant. In Group A, which had the highest survival rate, IgG3 levels were evaluated at different days postinfection. An elevation in IgG3 titre was observed on day 15 postinfection, followed by a decline by day 20 postinfection to levels comparable to the healthy unchallenged group (Figure 3(b)). These findings suggest that while *P. berghei* infection does not significantly induce IgG3 responses, probiotic treatment may modulate IgG3 levels during the course of infection.

#### 3.4.3. IFN-*γ*

The levels of IFN-*γ* were compared between all test groups and the healthy unchallenged group. The control group exhibited excessive production of IFN-*γ*. In contrast, Groups A, B, and C, which were fed laboratory-cultured yogurt, showed regulated IFN-*γ* levels (Figure 2(c)). Specifically, Group B displayed the next highest IFN-*γ* titre, whereas Group A and Group C had the lowest IFN-*γ* titres. These reductions were statistically significant. These findings suggest that prolonged administration of probiotics effectively controls the excessive production of IFN-*γ* in BALB/c mice infected with *P. berghei*. Furthermore, within Group A, IFN-*γ* titres were evaluated on days 10, 15 and 20 postinfection. An elevation in IFN-*γ* levels was observed on day 20 postinfection, which corresponded with higher parasitaemia values (Figure 3(c)). This correlation indicates that while prolonged probiotic administration mitigates excessive IFN-*γ* production, there is a notable increase in IFN-*γ* levels as parasitaemia intensifies towards the later stages of infection.

#### 3.4.4. IL-4

The treatment in Group A elicited the highest production of IL-4, followed by Group C (Figure 2(d)). Group B exhibited intermediate levels of IL-4, while the control group demonstrated the lowest IL-4 production. Within Group A, IL-4 levels were evaluated at different days postinfection. The highest IL-4 concentration was observed on day 10 postinfection; however, there was no significant difference in IL-4 levels between days 10 and 20 postinfection (*p* = 0.60) (Figure 3(d)). These findings suggest that prolonged administration of probiotics through formulated yogurt enhances IL-4 production in BALB/c mice infected with *P. berghei* ANKA. Nonetheless, the IL-4 response remains stable between days 10 and 20 postinfection, indicating a sustained immune modulation effect by the probiotic treatment.

## 4. Discussion

In this study, we used *L. fermentum* YZ01 to formulate yogurt that was fed to BALB/c mice infected with *P. berghei*, a rodent malaria parasite. Analysis of mouse faeces revealed an increase in the LAB population following yogurt consumption, suggesting significant modulation of the gut microbiota. This modulation likely enhanced the fermentation rate, increased lactic acid production, and decreased gut pH, creating an environment that supports the growth of beneficial bacteria [39].

Remarkably, groups treated with probiotics (Groups A, B and C) exhibited lower parasitaemia levels compared to the control group, potentially due to the antimicrobial properties of bacteriocins produced by LABs [40]. Similar findings have been reported with other LAB strains such as *Lactiplantibacillus plantarum*, which can produce antiplasmodial peptides that inhibit the growth of *P. falciparum* [41]. This suggests that *L. fermentum* YZ01 may produce similar bacteriocins that could inhibit *P. berghei* growth.

Prolonged consumption of probiotics also significantly enhanced the survival rates of the infected mice. For instance, by day 14 postinfection, Group A showed 0% mortality compared to 60% in the control group, and at the end of the experiment, the control group exhibited 100% mortality while Group A had only 40% mortality. These outcomes align with previous studies [23, 42] suggesting that gut microbiota modulation can substantially impact survival during *Plasmodium* infection. For example, Maegraith et al. [42] and Villarino et al. [23], showed that modulation of the gut microbiome affects the survival of experimental mice during *Plasmodium* infection. Maegraith et al. [42] observed suppression of *Plasmodium* development in *P. berghei* infected experimental groups of rats and mice that were fed with milk formulation. The *P. berghei* infected rats and mice were administered milk from bovine, Oster and normal diet. It was opined that there existed within the milk formulation some dietary factor that interfered with the erythrocytic stage of *Plasmodium* infection and probably prevented the death in groups of experimental animals fed with milk as against those on a normal diet. The unknown factor was later suggested by Villarino et al. [23] to be probiotics, which when fed to infected experimental mice increased their survival rate, an observation substantiated in this present study.

Furthermore, this study corroborates the significant influence of gut microbiota composition on the severity of malaria. The lowest levels of parasitaemia and enhanced survival rates in Group A, which had the most prolonged probiotic treatment, support the hypothesis that gut microbiota plays a crucial role in modulating disease progression. Although the specific factors that define the severity of malaria are still not fully understood and remain under investigation, parasitaemia is acknowledged as a pivotal indicator [43].

Compelling evidence from various studies supports the notion that variations in gut microbiota composition can significantly alter malaria severity. For instance, Taniguchi et al. [44] found that the severity of malaria, as manifested by cerebral and intestinal pathologies in *P. berghei* ANKA-infected C57BL/6 and BALB/c mice, varied according to the gut microbiota composition. This variation suggests that specific bacterial populations may either enhance resistance or increase susceptibility to malaria. Likewise, Fan et al. [45] identified a *Lactobacillus* sp. as the predominant gut microbiota species in healthy C57BL/6 mice infected with *P. berghei* ANKA, a finding echoing that of Villarino et al. [23], who noted a significant presence of *Bifidobacterium* and *Lactobacillus* in C57BL/6 and BALB/c mice infected with *Plasmodium yoelii.* This bacterial dominance is posited as a potential factor for reduced malaria severity due to a lower parasite burden in the experimental mice.

Further investigations have explored the relationship between gut microbiota variability and malaria resistance [24, 46]. Particularly, the transplantation of faecal microbiota that confers malaria resistance in pregnant mice resulted in a lower parasite burden, reduced malaria anaemia, and improved pregnancy outcomes, as reported by Morffy-Smith et al. [24]. This underscores the critical role of gut microbiota in influencing malaria severity.

However, contrasting findings by Omeiza et al. [47], where Swiss Albino mice infected with *P. berghei* ANKA were treated with Omidun—the supernatant from a fermented maize slurry containing 3 × 10^9^ CFU/mL of LAB—and achieved 100% parasite clearance, starkly differ from the absence of parasite clearance observed in this study. Moreover, Yooseph et al. [26] provided a compelling argument on the role of gut microbiota in human malaria infections. Their study revealed that *Streptococcus* and *Bifidobacterium* were the most prevalent species in individuals from malaria-endemic areas who exhibited a lower risk of *P. falciparum* infection, although no direct correlation was found between the gut microbiota composition and febrile malaria. These findings collectively highlight the complex interactions between gut microbiota and malaria pathogenesis, emphasizing the potential of targeting gut microbiota for therapeutic and preventive interventions in malaria.

The immune response analysis showed that the experimental groups had higher levels of IgM and reduced IFN-*γ* production compared to controls, supporting that probiotics may modulate immune responses favourably against malaria infection [23, 48]. While these immune factors may not directly target the malaria parasite, they likely interfere with the *Plasmodium* life cycle at critical stages—such as blocking sporozoite entry into liver cells, hindering the development of the erythrocytic stage and reducing transmission during the sexual stage of the parasite [49]. Therefore, the immune system plays a crucial role in parasite clearance, particularly through early-stage cytokine production. Additionally, the regulation of the adaptive immune system can prevent the overproduction of inflammatory cytokines, which could otherwise exacerbate disease severity [19, 50]. Previous studies have established that the levels of immune markers, like those measured in this study, are influenced by the composition of the microbiota [51].

IgM and IgG3 are essential at different phases of the immune response: IgM is one of the first antibodies produced following infection and plays a vital role in the initial containment of the malaria parasite. IgG3, conversely, is crucial for mediating antibody-dependent cellular cytotoxicity (ADCC) and activating the complement system, both of which are integral to the effective clearance of malaria-infected cells [15, 52]. The increased production of IgM observed in this study following administration of *L. fermentum* YZ01 aligns with findings from Yilmaz et al. [49], who reported enhanced IgM levels due to gut microbiota modulation. Additionally, Couper et al. [53] identified increased IgM production as a protective immune response against the asexual erythrocytic stage of *Plasmodium chabaudi* AS infection. Although this study found no significant differences (*p* = 0.413) in IgG3 levels among the groups, Ishih et al. [54] documented elevated IgG3 in both treated and untreated BALB/c mice infected with *P. berghei* NK65, suggesting variability in immune response among different experimental setups.

The production of IFN-*γ* is important in the control of *Plasmodium* infection, but excessive production of IFN-*γ* is detrimental as it can aggravate the severity of malaria [55]. In this study, there was an excessive production of IFN-*γ* in control infected mice while the level was lowered in the treated groups. When compared with the healthy unchallenged mice, the result indicated that the presence of probiotics in the gut controlled the excessive production of IFN-*γ* to almost the same level as found in uninfected mice. This suggests that the composition of LAB in the gut regulated IFN-*γ* production and that disease severity may be associated to the levels of IFN-*γ*. An increase in IFN-*γ* production has been reported to be responsible for early mortality in mice [54]. As noted by King and Lamb [55], IFN-*γ* is an important cytokine that controls *Plasmodium* infection in both exo-erythrocytic and erythrocytic stages of the life cycle of *Plasmodium* sp. In our study, the high levels of IFN-*γ* in the control infected mice may have resulted in the observed high mortality rate in this group.

It has been suggested that the balance between proinflammatory cytokines and anti-inflammatory cytokines such as IL-4 determines the level of severity in malaria while overproduction of any of the cytokines is also harmful [56]. In this study, increased production of IL-4 was observed among all the treated groups with group A having the highest titre while the control infected group has the least titre, lower than the healthy group. Consistent with Wu et al. [57], this study suggests IL-4 treatment in Group A mice reduces parasitaemia, promotes survival, and potentially mitigates experimental cerebral malaria, thereby extending lifespan. Additionally, the presence of alpha-gal modification in *Lactobacillus* [34], also modulated immune response mechanisms with protective capacity to pathogen infection [58].

In summary, this study highlights the significant role of *L. fermentum* YZ01 in modulating gut microbiota, which correlates with decreased parasitaemia and improved survival rates in BALB/c mice, underscoring a critical link between gut microbiota and malaria severity. The efficacy of *L. fermentum* YZ01 in producing bacteriocins or antimicrobial peptides suggests that such probiotics may not only inhibit the growth of malaria parasites but also positively alter host immune responses. The observed modulation of IgM and IFN-*γ* levels further supports the potential of probiotics to enhance systemic immune functions, which could mitigate the impacts of severe malaria. These findings advocate for the integration of probiotics into broader malaria management strategies and underline the importance of further research to dissect the precise mechanisms through which probiotics interact with the immune system and malaria pathogens.

## 5. Conclusions

This study substantiates the hypothesis that the gut microbiota significantly impacts malaria severity and progression. Utilizing BALB/c mice, we demonstrated that dietary intervention with *L. fermentum*-enriched yogurt modulates the gut microbiome, leading to reduced parasitaemia, enhanced survival rates, and a regulated immune response. These findings align with existing research suggesting that individuals with diverse gut microflora, often influenced by close association with nature or ethnic diets, may exhibit a natural immunity that mitigates the severity of diseases, including malaria. The protective effect observed in our mouse models reflects the potential of probiotics not only in managing gastrointestinal and respiratory infections but also in combating parasitic infections. Our results underscore the gut microbiota's role as a critical factor in the host's defence against malaria, mediated through dietary probiotics. Further biochemical investigations are warranted to isolate specific microbial interactions and molecular mechanisms that contribute to these protective effects, potentially paving the way for innovative therapeutic strategies against malaria. Future studies should aim to identify key molecules within probiotics that delay parasitaemia and enhance survival, offering new avenues for malaria treatment and prevention.

## Figures and Tables

**Table 1:** Experimental mice groups.

SN	Groups	Description
1	Group I: healthy group	Uninfected and untreated mice to monitor normal behavioural and physiological changes
2	Group II: control group	Negative control, infected and untreated mice to monitor parasitaemia progression
3	Group III: probiotics group A	3 weeks feeding with formulated yoghurt prior to infection
4	Group IV: probiotics group B	1 week feeding with formulated yoghurt prior to infection
5	Group V: probiotics group C	Treated with antibiotics (metronidazole) for 1 week followed by feeding with formulated yoghurt for 2 weeks prior to infection

## Data Availability

The data generated during this study are available upon reasonable request.
